# Ancestrality and Mosaicism of Giant Viruses Supporting the Definition of the Fourth TRUC of Microbes

**DOI:** 10.3389/fmicb.2018.02668

**Published:** 2018-11-27

**Authors:** Philippe Colson, Anthony Levasseur, Bernard La Scola, Vikas Sharma, Arshan Nasir, Pierre Pontarotti, Gustavo Caetano-Anollés, Didier Raoult

**Affiliations:** ^1^Aix-Marseille Université, Institut de Recherche pour le Développement (IRD), Assistance Publique – Hôpitaux de Marseille (AP-HM); Microbes, Evolution, Phylogeny and Infection (MEΦI); Institut Hospitalo-Universitaire (IHU) – Méditerranée Infection, Marseille, France; ^2^Centre National de la Recherche Scientifique, Marseille, France; ^3^Evolutionary Bioinformatics Laboratory, Department of Crop Sciences, University of Illinois Urbana-Champaign, Urbana, IL, United States; ^4^Department of Biosciences, COMSATS University Islamabad, Islamabad, Pakistan

**Keywords:** giant virus, TRUC, megavirales, mimivirus, informational genes, protein structural domains

## Abstract

Giant viruses of amoebae were discovered in 2003. Since then, their diversity has greatly expanded. They were suggested to form a fourth branch of life, collectively named ‘TRUC’ (for “Things Resisting Uncompleted Classifications”) alongside *Bacteria*, *Archaea*, and *Eukarya*. Their origin and ancestrality remain controversial. Here, we specify the evolution and definition of giant viruses. Phylogenetic and phenetic analyses of informational gene repertoires of giant viruses and selected bacteria, archaea and eukaryota were performed, including structural phylogenomics based on protein structural domains grouped into 289 universal fold superfamilies (FSFs). Hierarchical clustering analysis was performed based on a binary presence/absence matrix constructed using 727 informational COGs from cellular organisms. The presence/absence of ‘universal’ FSF domains was used to generate an unrooted maximum parsimony phylogenomic tree. Comparison of the gene content of a giant virus with those of a bacterium, an archaeon, and a eukaryote with small genomes was also performed. Overall, both cladistic analyses based on gene sequences of very central and ancient proteins and on highly conserved protein fold structures as well as phenetic analyses were congruent regarding the delineation of a fourth branch of microbes comprised by giant viruses. Giant viruses appeared as a basal group in the tree of all proteomes. A pangenome and core genome determined for *Rickettsia bellii* (bacteria), *Methanomassiliicoccus luminyensis* (archaeon), *Encephalitozoon intestinalis* (eukaryote), and Tupanvirus (giant virus) showed a substantial proportion of Tupanvirus genes that overlap with those of the cellular microbes. In addition, a substantial genome mosaicism was observed, with 51, 11, 8, and 0.2% of Tupanvirus genes best matching with viruses, eukaryota, bacteria, and archaea, respectively. Finally, we found that genes themselves may be subject to lateral sequence transfers. In summary, our data highlight the quantum leap between classical and giant viruses. Phylogenetic and phyletic analyses and the study of protein fold superfamilies confirm previous evidence of the existence of a fourth TRUC of life that includes giant viruses, and highlight its ancestrality and mosaicism. They also point out that best evolutionary representations for giant viruses and cellular microorganisms are rhizomes, and that sequence transfers rather than gene transfers have to be considered.

## Introduction

Since the Mimivirus discovery in 2003, dozens of giant viruses that infect *Acanthamoeba* spp. or *Vermamoeba vermiformis* have been isolated from various environmental samples, and more recently from animals including humans (La Scola et al., 2003; Raoult et al., 2004; Colson et al., 2017a). Currently, families *Mimiviridae* (La Scola et al., 2005) and *Marseilleviridae* (Boyer et al., 2009; Colson et al., 2013b) and isolates that represent new putative families of giant viruses of amoebae, including pandoraviruses (Philippe et al., 2013), pithoviruses (Legendre et al., 2015), faustoviruses (Reteno et al., 2015), Mollivirus (Legendre et al., 2015), Kaumoebavirus (Bajrai et al., 2016), cedratviruses (Andreani et al., 2016), Pacmanvirus (Andreani et al., 2017), and Orpheovirus (Andreani et al., 2018) have been described (Colson et al., 2017b). These giant viruses of amoebae exhibit unique phenotypic and genotypic characteristics that differentiate them from ‘traditional’ viruses and bring them close to small microbes (Lwoff, 1957; Colson et al., 2017a).

These viruses were linked through phylogenomic analyses to poxviruses, asfarviruses, ascoviruses, iridoviruses, and phycodnaviruses (formerly the largest viral representatives), which were grouped in 2001 in a superfamily named nucleocytoplasmic large DNA viruses (NCLDVs) (Iyer et al., 2001, 2006; Raoult et al., 2004). NCLDVs and giant viruses of amoebae were reported to share a putative ancient common ancestor harboring about 50 conserved core genes responsible for key viral functions (Yutin et al., 2009; Koonin and Yutin, 2010; Yutin and Koonin, 2012). Together with a common virion architecture and common major biological features including reproduction within cytoplasmic factories, this contributed to propose reclassifying NCLDVs, mimiviruses and marseilleviruses in a new viral order named Megavirales (Colson et al., 2013a).

The origin and ancestrality of giant viruses has remained controversial. From the onset, when the Mimivirus genome was sequenced in 2004, a phylogeny based on seven concatenated universally conserved genes showed that Mimivirus branched near the origin of the eukaryotic branch, and it was suggested that giant viruses comprised a fourth additional branch in the Tree of Life, alongside *Bacteria*, *Archaea*, and *Eukarya* (Raoult et al., 2004). This hypothesis was thereafter strengthened by both cladistic and phenetic analyses based on informational genes, including those implicated in nucleotide biosynthesis, transcription and translation (Boyer et al., 2010). The hypothesis of the existence of a fourth branch of microbes prompted to define the ‘TRUCs,’ which is an acronym for “Things Resisting Uncompleted Classifications” (Raoult, 2013, 2014). This term was coined because the definition of domains of life by C.R. Woese was based on ribosomal genes that are absent in giant viruses. This proposal of a fourth branch of life comprised by giant viruses has remained controversial and a subject of debate among virologists and evolutionary biologists. Some phylogenetic analyses were deemed to suggest complex patterns of evolutionary relationships for different informational proteins from giant viruses, which even questioned the monophyly of NCLDVs (Yutin and Koonin, 2012; Yutin et al., 2014). A high level of mosaicism has been highlighted for the genomes of giant viruses of amoebae, which was related to sequence transfers with organisms belonging to the three cellular domains of Life (Raoult et al., 2004; Boyer et al., 2009). A substantial gene flow has been also described in NCLDVs including in coccolithoviruses (Wilson et al., 2009; Nissimov et al., 2017). It was suspected that lateral gene transfers blurred phylogenies based on genes shared by giant viruses and cellular organisms (Moreira and Lopez-Garcia, 2009). Several phylogenetic reconstructions in which giant viruses branch within eukaryotes were published (Moreira and Lopez-Garcia, 2009, 2015; Williams et al., 2011), and it was put forward that the universally conserved genes used in phylogeny reconstructions might have been acquired by giant viruses from their proto-eukaryotic hosts (Moreira and Lopez-Garcia, 2009; Yutin et al., 2014). The interpretation of some phylogenies was also that modern giant viruses might originate from smaller NCLDVs (Yutin and Koonin, 2013; Yutin et al., 2014). Conversely, it was proposed that giant viruses might derive from ancestral cellular genomes by reductive evolution (Legendre et al., 2012). Besides, phylogenetic reconstructions supporting the fourth TRUC hypothesis triggered methodological criticisms arguing that they were distorted by long-branch attraction and technical issues, and divergences in their interpretation. However, alternative phylogenies were not accurate either regarding the phylogeny of *Archaea*, *Bacteria*, or *Eukarya* (Williams et al., 2011; Moreira and Lopez-Garcia, 2015). A four-branch topology was also obtained by reconstructing phylogenies that describe the evolution of proteomes and protein domain structures (Nasir et al., 2012; Nasir and Caetano-Anollés, 2015). The genomic and structural diversity embedded in giant virus proteomes was found similar to that of proteomes of cellular organisms with parasitic lifestyles. Beyond, other phylogenies based on RNA polymerase suggested the presence in metagenomes of sequences related to giant virus relatives (Wu et al., 2011; Sharma et al., 2014). As a synthesis, it was deemed that more work is needed on Megavirales phylogenies to clarify if these viruses are monophyletic or have different evolutionary histories (Forterre and Gaia, 2016). Here, we specify the definition of giant viruses, highlight their mosaicism at the genome, structure and sequence level, and strengthen the evidence for their ancestrality and the existence of a fourth TRUC of microbes.

## Materials and Methods

### Definition of Giant Viruses

We collected and reviewed current knowledge on giant viruses from articles gathered from the NCBI PubMed database and from Google Scholar using as keywords “giant virus”; Megavirales; mimivir^∗^; marseillevir^∗^; pandoravir^∗^; pithovir^∗^; faustovir^∗^; mollivirus; cedratvirus; kaumoebavirus; pacmanvirus; virophage; transpoviron. We then compared the phenotypic and genotypic features of these viruses with those used as criteria to define classical viruses and those that are hallmark features of cellular organisms. The list of those criteria is presented in Table 1.

**Table 1:** Comparison of major features used as criteria to define classical viruses with those of giant viruses and to hallmark features of cellular microbes.

Phenotypic and genotypic characteristics	Classical viruses		Giant viruses		Cellular micro-organisms	
	Majority case	Exceptions/comments	Majority case	Exceptions/comments	Majority case	Exceptions/comments
Visible under a light microscope (>0.2 μm)	No	–	Yes	–	Yes	–
Genome size > 350 kbp	No		Yes	–	Yes	–
Presence of a virally-encoded capsid	Yes	Some capsidless viruses: genus *Mitovirus*, *Umbravirus*, *Hypovirus*, *Endornavirus* (Koonin and Dolja, 2014)	Yes	Pandoraviruses (Abergel et al., 2015)	No	Icosahedral compartments exist in bacteria and archaea that resemble to viral capsids: the encapsulin nanocompartments structurally similar to and possibly derived from major capsid proteins of tailed bacterial and archaeal caudaviruses, and bacterial microcompartments present in bacteria (including cyanobacteria and many chemotropic bacteria) that encapsulate enzymes involved in metabolic pathways (Tanaka et al., 2008; Krupovic and Koonin, 2017)
Presence of DNA and RNA inside the viral particle	No	Cytomegalovirus (Terhune et al., 2004)	Yes	–	Yes	–
Absolute parasitism	Yes	–	Yes	–	Several bacteria and archeae	Case of strictly intracellular microorganisms
Multiplication by binary fission	No	–	No	–	Yes	No *bona fide* binary fission for *Chlamydia* spp. (Abdelrahman et al., 2016; Bou Khalil et al., 2016), *Ehrlichia* spp. (Zhang et al., 2007), and *Babela* sp. (Pagnier et al., 2015)
Eclipse period during the replicative cycle	Yes	–	Yes	–	No	–
Entry into host cells by phagocytosis	No	–	Yes	–	–	–
Presence of a virus factory	In several viruses (e.g., adenoviruses, polyadenoviruses) (Netherton and Wileman, 2011)	–	Yes	Mollivirus (Legendre et al., 2015)	–	Morula similar to a viral factory for *Chlamydia* spp. (Abdelrahman et al., 2016; Bou Khalil et al., 2016), *Ehrlichia* spp. (Zhang et al., 2007), and *Babela* sp. (Pagnier et al., 2015)
Energy (ATP) generating machinery	No	–	No	–	Yes	Through glycolysis in *Mycoplasma genitalium* (Glass et al., 2006); absence in *Carsonella ruddii* (Nakabachi et al., 2006)
Presence of genes encoding ribosomal RNA and proteins	No	–	No	–	Yes	Uncomplete sets of ribosomal proteins and aminoacyl-tRNA synthetase in *Carsonella ruddii* (Tamames et al., 2007)
Presence of genes encoding translation-associated proteins	No	–	Yes	–	Yes	–
Presence of tRNA genes	No	–	Yes	Marseilleviruses, faustoviruses (Reteno et al., 2015)	Yes	–
Presence of viral proteins of transcription	Yes	–	Yes	Not detected by proteomics in a marseillevirus (Fabre et al., 2017)	Yes	–
Presence of host ribosomal proteins inside virions	No	Arenaviruses (Baird et al., 2012)	In Mollivirus (Legendre et al., 2015)	–	Yes	–
Presence of group I, II or spliceosomal introns, inteins	No	–	Yes	–	Yes	–
Transposable elements	No	–	Yes	Introns, inteins, transpovirons, miniature inverted-repeat transposable elements (MITEs, in pandoraviruses)	Yes	–
Infection by other viruses	No	–	No	Mimiviruses with (pro)virophages (La Scola et al., 2008; Fischer and Suttle, 2011)		
Mechanism of defense against viruses	No	–	Yes for mimiviruses	–	Yes	–
High level of genome mosaicism	No	–	Yes	–	Yes	–
Evidence of ancestrality based on conserved/ubiquitous genes and protein fold-superfamilies	Four monophyletic classes of viruses (Koonin et al., 2006)	–	Yes	–	Yes	–

### Protein Structure Assignment to Viral and Cellular Proteomes

Protein sequences from completely-sequenced proteomes of 80 Megavirales were scanned against the library of hidden Markov models (HMMs) of structural recognition maintained by the SUPERFAMILY database for structure assignment at an *E*-value cutoff of < 0.0001 (Gough et al., 2001; Gough and Chothia, 2002). The SUPERFAMILY HMMs represent proteins of known three-dimensional (3D) structures and assign each detected occurrence of protein domain into fold superfamilies (FSFs), as defined by the Structural Classification of Proteins (ver. 1.75) database (Andreeva et al., 2008). FSFs are collections of one or more protein families that show recognizable 3D structural and functional similarities, but not necessarily sequence identities, that are indicative of common origin. Thus, FSFs represent highly dissimilar protein domains at the sequence level that have evolved via divergence from a common structure and can still be recognized based on the presence of that conserved structural core by HMMs trained to detect remote homologies. Because of the fast mutation rates of viral genes, it sometimes becomes impossible to generate meaningful global sequence alignments when considering viral and cellular genes together in data matrices. The fast mutation rates, especially when considering proteins separated by large evolutionary distances and involving distantly related taxa, lead to alignment inaccuracies and large number of gaps. In contrast, protein structure evolves at least 3 to 10 times slower than molecular sequences (Illergard et al., 2009) and hence provides an alternative to study the deep evolutionary history of cells and viruses (Nasir et al., 2012; Nasir and Caetano-Anollés, 2015). In parallel, FSF assignments for a total of 102 cellular organisms including an equal number of archaea, bacteria, and eukaryota were retrieved from a previous work during which a total of 1,797 distinct FSF domains had been detected (*E*-value < 0.0001) (Nasir and Caetano-Anollés, 2015).

### Structural Phylogenomics

Using an in-house Python script, we generated a data matrix containing 182 rows (proteomes from 34 archaea, 34 bacteria, 34 eukaryota, and 80 Megavirales members) and 289 columns (FSFs) containing presence/absence information for ‘universal’ FSFs. ‘Universal’ FSFs, by definition, included FSFs that were detected in at least one proteome each from archaea, bacteria, eukaryota, and a Megavirales member. In other words, FSFs unique to one of these four groups (e.g., bacteria-specific FSFs) or shared by 2-to-3 groups of cellular organisms and/or viruses (e.g., FSFs detected in archaea, bacteria, and viruses but not eukaryota) were excluded from our definition of universal FSFs (see (Nasir et al., 2015) for details on FSF groups in cellular organisms and viruses). This data matrix containing 182 proteomes and 289 universal FSFs was imported into the PAUP (ver. 4.0b10) software (Swofford, 2002) for phylogenomic tree reconstruction. Proteomes were treated as taxa and FSFs as characters. Presence/absence of FSFs (represented by 1 and 0, respectively) were used as distinct character states to distinguish taxa. Maximum parsimony method was set as optimality criterion to reconstruct the most parsimonious unrooted phylogenomic tree describing the evolution of sampled proteomes based on the presence/absence of 286 parsimony informative FSF characters. The unrooted reconstructed tree was rooted *a posteriori* by the branch resulting in minimum increase in overall tree length using the Lundberg method (Lundberg, 1972; see Nasir et al., 2017; Caetano-Anollés et al., 2018 for description and review of rooting methodology). The reliability of the phylogenetic splits was evaluated by running 1,000 bootstraps. Separately, we performed principal coordinate analysis (PCoA) on the same data matrix and plotted the 182 sampled viral and cellular proteomes into 3D space. Proteomes are composed of FSF domains of different evolutionary and geological ages. From a previously reconstructed tree of domains (ToD) (Nasir and Caetano-Anollés, 2015), we retrieved the relative evolutionary ages for each of the 289 universal FSFs. The relative scale reflects the distance of each node (FSF domain) from the root of the ToD and ranges from 0 (closer to the root, most ancient) to 1 (most recent). The node distance (*nd*) value thus describes a clock-like behavior for the evolution of FSF domains and has previously been linked to the geological record (Wang et al., 2011). Euclidean distance was used to plot proteome dissimilarity based on the 1-*nd* transformation of the *nd* scale for each FSF domain in every proteome, as previously (Nasir and Caetano-Anollés, 2015). Since the PCoA is centered around *nd* variable derived from an evolutionary tree, we refer to this method as evo-PCoA. The evo-PCoA thus projects proteome dissimilarity into 3D space based on differences in the evolutionary ages of components of each proteome. XLSTAT plugin was added to Microsoft Excel for generation of PCoA.

### Collection of Orthologous Sequences From Viruses

Analysis was performed as described in previous works (Boyer et al., 2010; Sharma et al., 2014). The genes used in the present study were identified from clusters of orthologous groups of proteins (COGs) involved in nucleotide transport and metabolism and information storage and processing (i.e., categories F, J, A, K, L, and B). These genes comprise proteins that are the most conserved between cellular organisms and viruses (Boyer et al., 2010). They notably include three genes conserved among previously identified *Megavirales* representatives and in faustoviruses, and that encode DNA-dependent RNA polymerase subunits 1 (RNAP1) and 2 (RNAP2), and family B DNA polymerase (DNApol). Viral orthologs for these three genes were retrieved with the OrthoMCL program (Li et al., 2003) from the gene complements of 317 viral genomes harboring > 100 genes downloaded from the NCBI sequence databases^1^, and orthologs from nine faustovirus genomes (Benamar et al., 2016) and Mollivirus sibericum (Legendre et al., 2015) were added to this sequence set (Supplementary Table S1).

### Collection of Orthologous Sequences From Cellular Organisms

Informational gene homologs from cellular organisms (maximum number: 20,000) were retrieved from the NCBI GenBank non-redundant (nr) protein sequence database by stand-alone BLAST searches with viral sequences as query, using default parameters except for the maximum target number limit, set to 20,000 (Altschul et al., 1990). Homologous sequences were selected from representative species that diverged approximately 500 million years ago using TimeTree (Hedges et al., 2006; Sharma et al., 2014). BLASTp results were filtered by taxon identifiers, selected sequences were downloaded using their GenBank identifier, and duplicates were removed by clustering with the CD-HIT suite, as previously described (Sharma et al., 2014, 2015b).

### Multiple Sequence Alignments and Phylogeny Reconstructions

Sequences (Supplementary Table S2) were aligned with the MUSCLE software (Edgar, 2004) and alignments were manually curated. Phylogeny reconstructions were performed using FastTree (Price et al., 2010) with the Maximum Likelihood method, and the CAT 20 model that analyses the alignment site by site and reduces long branch attraction artifacts (Lartillot et al., 2007). Then, trees were visualized using FigTree^2^. Confidence values were determined by the Shimodaira-Hasegawa (SH) test using FastTree (Price et al., 2010).

### Comparison of Informational Genes Repertoires

Hierarchical clustering was performed with the Pearson distance method and the TM4 multi-package software, as previously described (Sharma et al., 2015a,b). This analysis relied on the comparison of the presence/absence patterns of 726 COGs involved in nucleotide transport and metabolism and information storage and processing in the gene contents of viruses and of selected bacterial, archaeal, and eukaryotic representatives (Sharma et al., 2015a,b). Viral orthologs were identified through BLASTp searches using these 726 COGs. BLAST searches were performed with default parameters, except for the maximum target number limit, set to 20,000.

### Comparison of Gene Repertoires From a Representative of Each of the Three Cellular Domains of Life and From a Giant Virus, and Construction of the Rhizome of Genomes and Genes

Comparison of the gene contents was performed for three members of cellular domains that were selected because they harbor small genomes and are intracellular parasites [namely *Encephalitozoon intestinalis* (an eukaryote) (Corradi et al., 2010), *Methanomassiliicoccus luminyensis* (an archaeon) (Gorlas et al., 2012), *Rickettsia bellii* (a bacterium) (Ogata et al., 2006)], and for Tupanvirus soda lake (Abrahao et al., 2018), a recently described giant virus that was selected here because it has a particularly large gene content and harbors the largest set of translation components among giant viruses. This comparison used the ProteinOrtho v5 tool with 1e-3, 20 and 30% as thresholds for e-value, amino acid identity, and coverage of aligned sequences, respectively (Lechner et al., 2011). In addition, best BLASTp hits against the NCBI GenBank protein sequence database were obtained for these four organisms. The “rhizomes” of the genomes were built using the Circos tool^3^. Rhizomes consist in a representation of the genome evolution and mosaicism that takes into consideration the fact that genes from this genome as well as intragenic sequences do not have the same evolutionary history, and can result from exchanges, fusions, recombination, degradation, or *de novo* creation (Raoult, 2010). Rhizomes, which are devoid of a center, were proposed as a better paradigm of genetic evolution than trees (Deleuze and Guattari, 1976; Raoult, 2010). Rhizomes built here show in a single figure, for all the genes from a given virus or cellular organisms, the taxonomy of their best BLASTp hits that represent putative donors or acceptors involved in sequence transfers, as well as the ORFans (sequences devoid of homolog in databases). Furthermore, a rhizome of genes was also determined for the genes encoding a methionyl-tRNA synthetase shared by the four organisms, by performing BLASTp searches with fragments obtained from this gene by cutting its amino acid sequence into 40 amino acid-long fragments that overlapped with a sliding window of 20 amino acids.

## Results and Discussion

### Phylogenetic Analyses of Protein Structural Domains of Viral and Cellular Proteomes

A total of ∼1,200 folds, ∼2,000 superfamilies, and ∼5,000 families of structural domains encompass the entire evolutionary and functional diversity of the protein world. The history of these folds, superfamilies and families has been traced with phylogenomic methods by studying the entire repertoires of proteins (proteomes), beginning with a study of a small set of 32 completely sequenced genomes (Caetano-Anollés and Caetano-Anollés, 2003) and continuing with a recent extended analysis of thousands of viral and cellular genomes (Nasir and Caetano-Anollés, 2015). Timelines of domain history could be calibrated with a molecular clock that relates them to the geological record (Wang et al., 2011). The timelines showed that the oldest domain families harbored ‘Rossmann-like’ α/β/α-layered and bundle structures typical of globular proteins, followed by barrel structures typical of membrane and metabolic proteins (Caetano-Anollés et al., 2012). The oldest of these structures are predominant in membrane-associated proteins, suggesting a very early onset of cellular structure. Their link to metabolism, but not translation, also suggests the late development of the genetic code and the late appearance of the ribosome (Harish and Caetano-Anollés, 2012; Caetano-Anollés et al., 2013). Remarkably, the late arrival of modern genetics ∼3 billion years (Gy) ago signals the end of a period responsible for the primordial cellular origin of viruses, clearly evident by the fact that the oldest superfamilies are common to cells and viruses (Nasir and Caetano-Anollés, 2015). In addition, these data also indicated that RNA polymerases are more ancient than the ribosome. Such diversification occurred prior to the appearance of the cellular domains of life.

**FIGURE 1:**
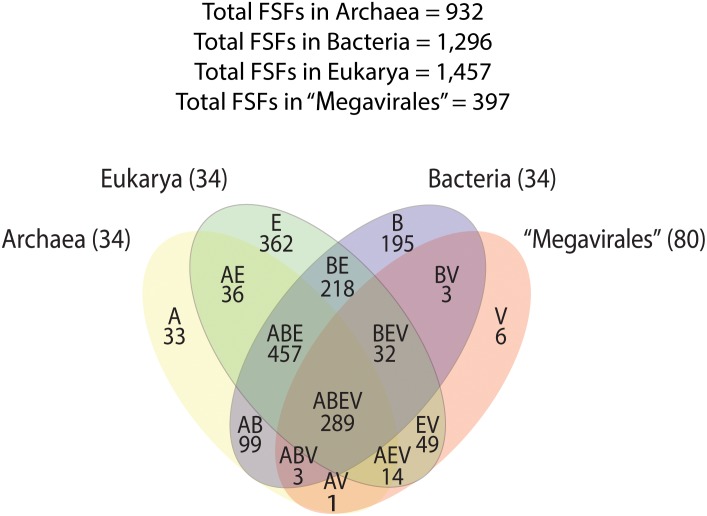
Venn diagram displaying FSF distribution and sharing patterns among *Archaea*, *Bacteria*, *Eukarya*, and Megavirales. A, *Archaea*; B, *Bacteria*; E, *Eukarya*; FSF, fold superfamilies; V, viruses.

**FIGURE 2:**
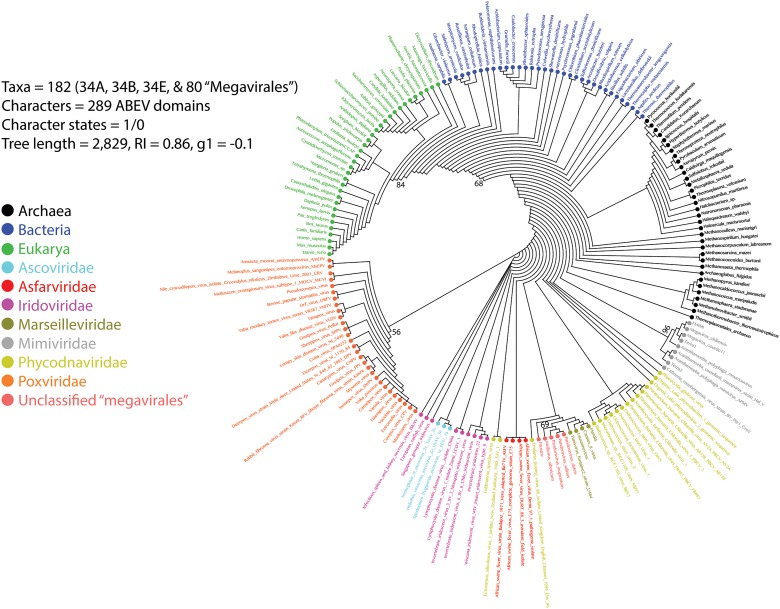
Phylogeny of proteomes describing the evolution of 182 proteomes randomly sampled from cellular organisms and viruses. The universal Tree of Life is rooted using Weston’s generality criterion. The 102 cellular proteomes are from Nasir and Caetano-Anollés (2015).

**FIGURE 3:**
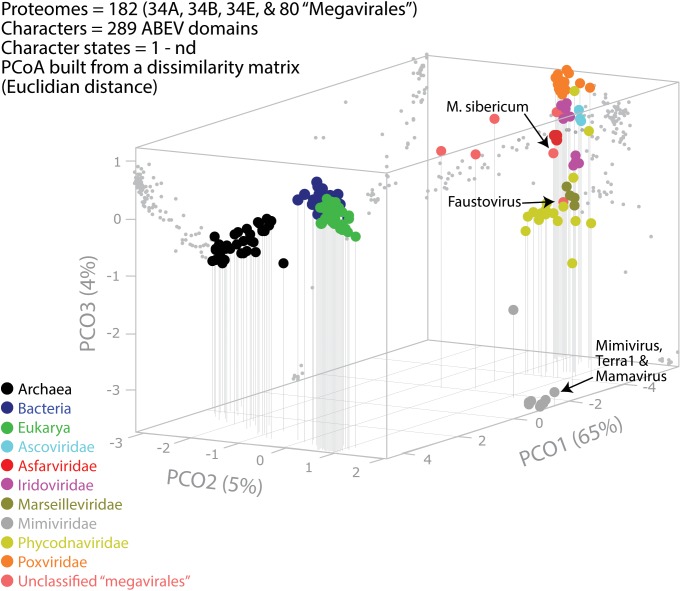
Evolutionary principal coordinate (evoPCO) analysis plot portrays in its first three axes the evolutionary distances between cellular and viral proteomes. The percentage of variability explained by each coordinate is given in parentheses on each axis. Data points of the 3-dimensional scatter plot describing temporal clouds are mapped onto projections planes and connected with vertical leading drop lines along the PCO3 axis. The list of whole coordinate information for building the PCoA plot of this figure is provided in Supplementary Table S3.

**FIGURE 4:**
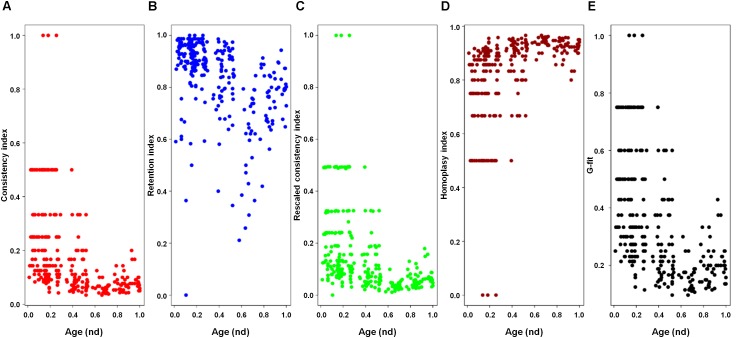
Plots of the indices of the phylogenetic tree of proteomes describing the evolution of 182 proteomes randomly sampled from cellular organisms and viruses (corresponding to Figure 2) against the age of the phylogenetic character [fold superfamily (FSF)]. Five measures of the levels of lateral sequence transfers for the maximum parsimony tree reconstruction performed in the present study, namely consistency index **(A)**, retention index **(B)**, rescaled consistency index **(C)**, homoplasy index **(D)**, and G-fit **(E)**, are plotted against the age of the phylogenetic character FSF [measured as node distance (*nd*) values] for 289 characters (FSF) shared by archaea, bacteria, eukaryota, and viruses. High retention indices, especially for lower *nd* values (corresponding to older domains), indicates excellent fit of the characters to the phylogeny.

A previous phylogenomic data-driven analysis of proteomes confirmed the early cellular origin of viruses and the rise of viral RNA proteomes followed by that of DNA viruses and Megavirales representatives (Nasir and Caetano-Anollés, 2015). Here we focused on the evolutionary relationship of Megavirales and cellular organisms. Out of all possible FSF domains (Figure 1), we selected 289 that were universal, i.e., that were shared by viruses and cellular organisms. We then used this set to build a phylogeny of proteomes (Figure 2). Megavirales representatives appear as a basal group in the tree of proteomes, which is consistent with results from sequence analyses performed here and previously (Boyer et al., 2010; Sharma et al., 2014). The subgroup that was closest to cellular organisms was family *Mimiviridae*, followed by family *Phycodnaviridae* and then groups comprised by family *Marseilleviridae* and by faustoviruses, mollivirus, and pandoraviruses. Similar phylogenetic patterms were revealed when we used multidimensional scaling approaches to explore the temporal space of ages of individual structural domains in proteomes (Figure 3). We found distinct temporal clouds of proteomes for viruses and organisms belonging to *Archaea*, *Bacteria*, and *Eukarya.* The *Mimiviridae* group was clearly dissected from the main viral cloud, which was temporally closer to cellular proteomes, suggesting their late appearance in viral evolution. Again, the family *Phycodnaviridae* appeared between the family *Mimiviridae* and the rest of the viral cloud. In terms of the proportions of FSFs detected in giant viral groups, asfarviruses have a proteome that is more similar to that of faustovirus, which is consistent with phylogenetic analysis of sequences. However, when considering raw number, mimiviruses have more FSFs in common with faustovirus. Finally, when plotting phylogenetic indices measuring the levels of homoplasy of the MP tree reconstruction (corresponding to Figure 2) against age of the phylogenetic character (fold superfamily), high retention indices, especially for lower *nd* values (oldest domains), indicated excellent fit of characters to the phylogeny (Figure 4). Homoplasy indicates the level of independent gain of characters in lineages and is a good indicator of deviations from vertical inheritance (Farris, 1983). The levels of homoplasy were moderate for protein folds, showing that the vertical signals override the horizontal signals.

### Phylogenetic Analyses of RNA and DNA Polymerases and Phenetic Comparison of Informational COGs

**FIGURE 5:**
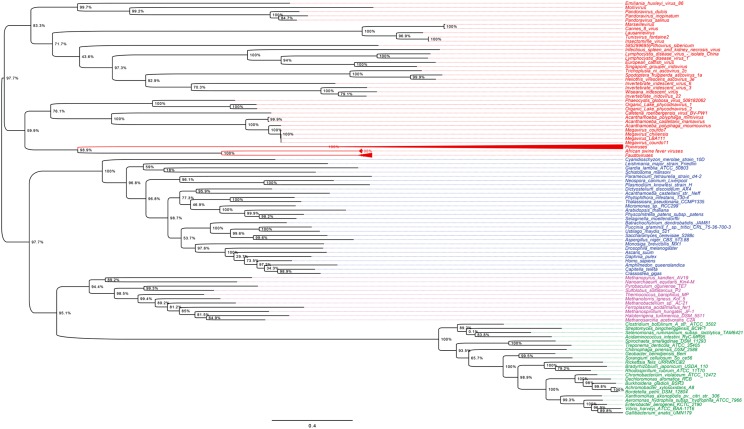
RNAP1 phylogenetic tree. The RNAP1 tree was built by using aligned protein sequences from *Megavirales* (red), *Bacteria* (green), *Archaea* (pink), and *Eukarya* (blue). Confidence values were calculated by the Shimodaira-Hasegawa (SH) test using the FastTree program (Price et al., 2010). Average length of sequences was 1,336 amino acids. The scale bar represents the number of estimated changes per position.

**FIGURE 6:**
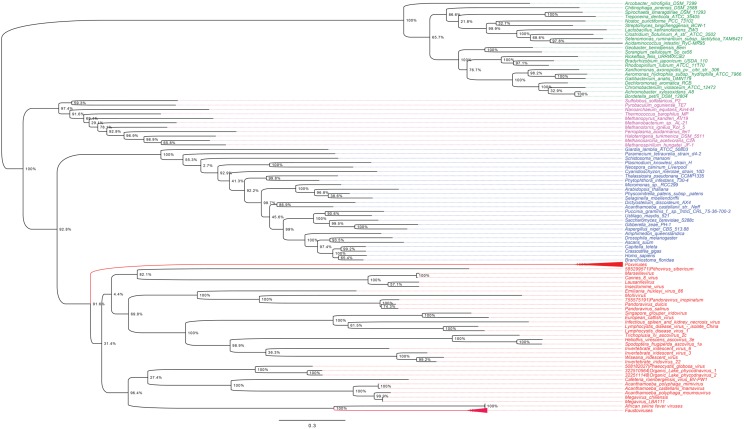
RNAP2 phylogenetic tree. The RNAP2 tree was built by using aligned protein sequences from *Megavirales* (red), *Bacteria* (green), *Archaea* (pink), and *Eukarya* (blue). Confidence values were calculated by the SH test using the FastTree program (Price et al., 2010). Average length of sequences was 1,188 amino acids. The scale bar represents the number of estimated changes per position.

**FIGURE 7:**
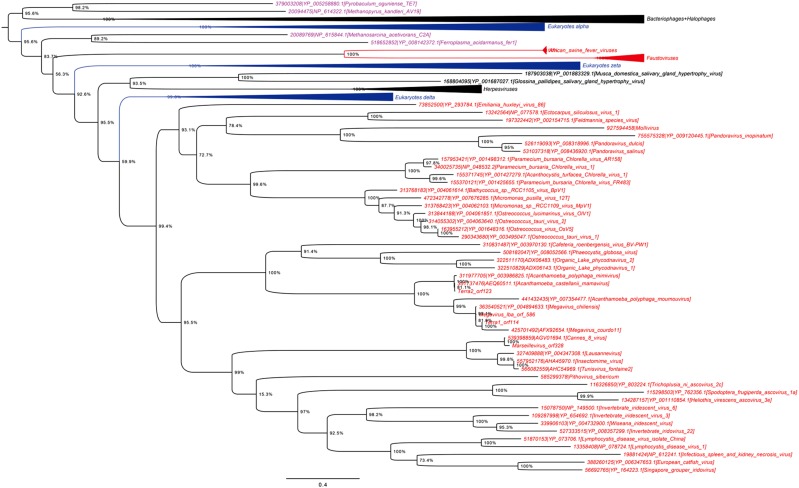
DNA polymerase phylogenetic tree. The DNA polymerase tree was built by using aligned protein sequences from *Megavirales* (red), *Bacteria* (green), *Archaea* (pink), and *Eukarya* (blue). Confidence values were calculated by the SH support using the FastTree program (Price et al., 2010). Average length of sequences was 1,134 amino acids. The scale bar represents the number of estimated changes per position.

**FIGURE 8:**
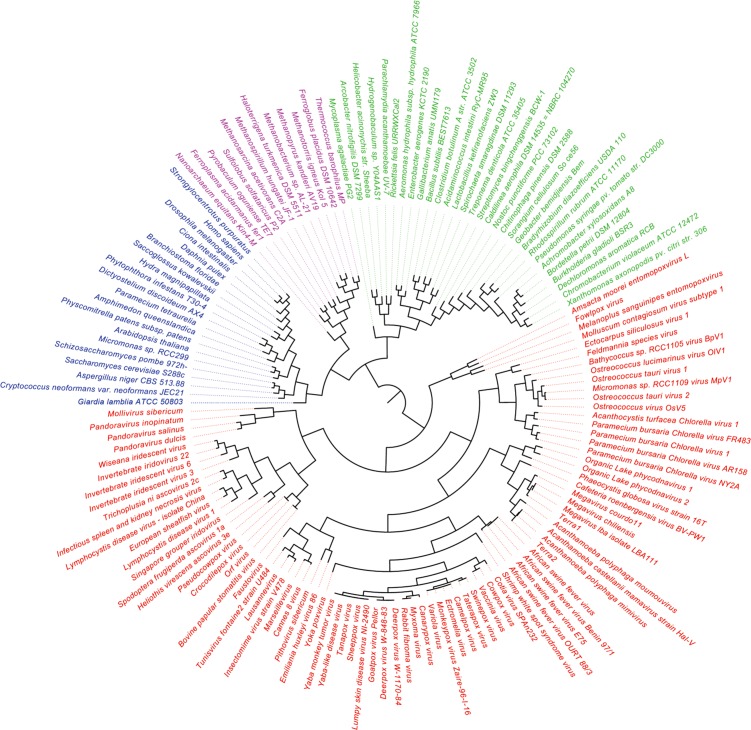
Hierarchical clustering by phyletic pattern based on the presence/absence of informational Clusters of Orthologous Groups (COGs) of proteins. The Megavirales members are represented in red, *Bacteria* members in green, *Archaea* members in pink, and *Eukarya* members in blue.

As shown in Figures 5, 6, trees reconstructed using both RNA polymerase subunit sequences (RNAP1 and 2) from members of Megavirales (including recently described giant viruses of amoebae), *Bacteria*, *Archaea*, and *Eukarya* clearly displayed a topology with four branches. The Megavirales group exhibits a considerable genetic diversity. Regarding phylogeny reconstruction based on DNA polymerases present in archaea, eukaryotes and giant viruses, giant viruses are separated into two groups. Faustoviruses and asfarviruses are clustered together and comprise sister branches, apart from other giant viruses that form an independent and strongly supported cluster (Figure 7). Hierarchical clustering analysis was performed based on a binary presence/absence matrix constructed using 727 informational COGs present in 143 representative genomes of cellular organisms from *Bacteria*, *Archaea* and *Eukarya*, and viruses from Megavirales (Figure 8). This phenetic analysis based on informational genes also showed a four-branch topology, Megavirales being a distinct branch alongside *Eukarya*, *Archaea*, and *Bacteria*.

### Pangenome and Core Genome for One Member of Each of the Three Cellular Domains of Life and of a Giant Virus

A pangenome and core genome was determined for one representative of each of the four TRUCs of microbes: namely *R. bellii* (bacteria, 1,430 genes), *M. luminyensis* (archaea, 2,533 genes), *E. intestinalis* (eukaryota, 1,910 genes), and Tupanvirus soda lake (giant virus, 1,269 genes). The pangenome describes the full complement of genes in a group of organisms, in our case the four microbes, and is comprised by the core genome that contains genes present in all 4 microbes and by the dispensable genome composed of genes that are unique to each microbe and genes absent from one or more microbes. The pangenome of these four microbes was composed of 6,531 genes, and their core genome (shared by all four organisms) was composed of 33 genes that represented between 1.3 and 2.6% of their gene contents. This core genome included notably genes encoding a DNA-directed RNA polymerase, a ribonucleoside-diphosphate reductase, a translation elongation factor 2, and several aminoacyl-tRNA synthetases. A majority of these genes therefore consisted of translation components. In addition, 23 (1.6%), 68 (5.4%), 13 (0.7%), and 68 (5.4%) genes from *R. bellii*, *M. luminyensis*, *E. intestinalis*, and Tupanvirus, respectively, had homologs in the genomes of two other microbes. Finally, 261 genes in *R. bellii* (18.3%), 362 in *M. luminyensis* (14.3%), 298 in *E. intestinalis* (15.6%), and 132 in Tupanvirus (10.4%) had homologs in at least one of the three other microbes. These results show that beyond the fact that the number of genes for Tupanvirus is in the same order of magnitude than for the three cellular microorganisms, a substantial proportion of the genes of this giant virus overlaps with those of the bacteria, the archaeon and the eukaryote.

### Rhizomes of Genomes and Genes as Appropriate Representations of the Origin and Evolution of Members From the Four TRUCs of Microbes

**FIGURE 9:**
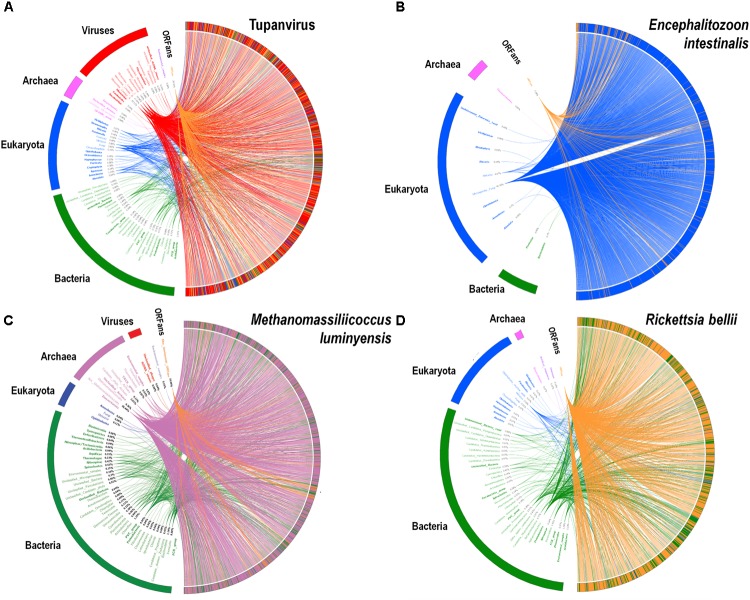
Rhizomes of genomes illustrative of the mosaicism of the genomes of representatives of the four TRUCs of microbes including Tupanvirus soda lake (a mimivirus) **(A)**; *Encephalitozoon intestinalis* (a microbial eukaryote) **(B)**; *Methanomassiliicoccus luminyensis* (an archaeon) **(C)**; and *Rickettsia bellii* (a bacterium) **(D)**. The genes of these four microorganisms were linked to their most similar sequences in the NCBI GenBank protein sequence database according to the BLAST program (https://blast.ncbi.nlm.nih.gov/Blast.cgi), classified according to their belonging to viruses, eukaryotes, bacteria or archaea, and integrated in a circular gene data visualization. The figures were performed using the CIRCOS online tool (http://mkweb.bcgsc.ca/tableviewer/visualize/). Circular representations in **A** and **C** are the same than those produced for figures from articles Abrahao et al. (2018) and Levasseur et al. (2017), respectively, as they originate from the same data. These representations are licensed under CC BY 4.0 (https://creativecommons.org/licenses/by/4.0/) and CC-BY-NC (https://creativecommons.org/licenses/by-nc/4.0/), respectively.

**FIGURE 10:**
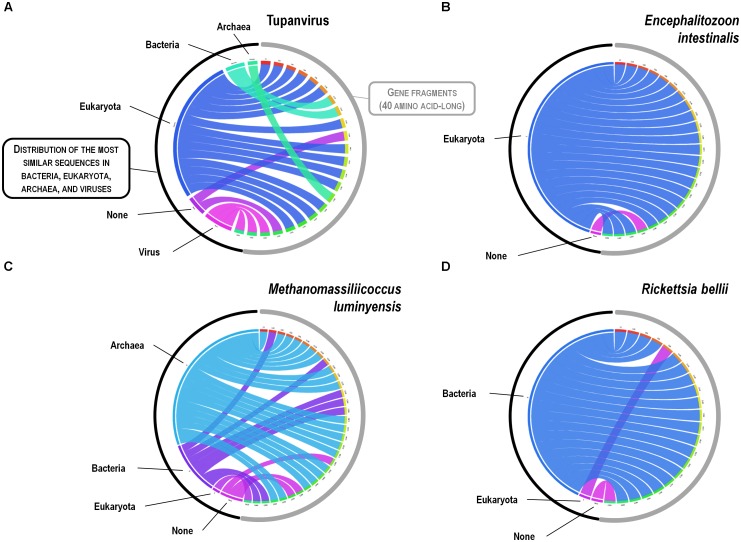
Rhizomes of methionyl-tRNA synthetase gene fragments illustrative of the mosaicism of the genes of representatives of the four TRUCs of microbes including Tupanvirus soda lake (a mimivirus) **(A)**; *Encephalitozoon intestinalis* (a microbial eukaryote) **(B)**; *Methanomassiliicoccus luminyensis* (an archaeon) **(C)**; and *Rickettsia bellii* (a bacterium) **(D)**. Forty amino acid-long fragments of the methionyl-tRNA synthetase encoding genes of the four microorganisms were linked to their most similar sequences in the NCBI GenBank protein sequence database according to the BLAST program (https://blast.ncbi.nlm.nih.gov/Blast.cgi), classified according to their belonging to viruses, eukaryotes, bacteria or archaea, and integrated in a circular gene data visualization. The figures were performed using the CIRCOS online tool (http://mkweb.bcgsc.ca/tableviewer/visualize/).

**FIGURE 11:**
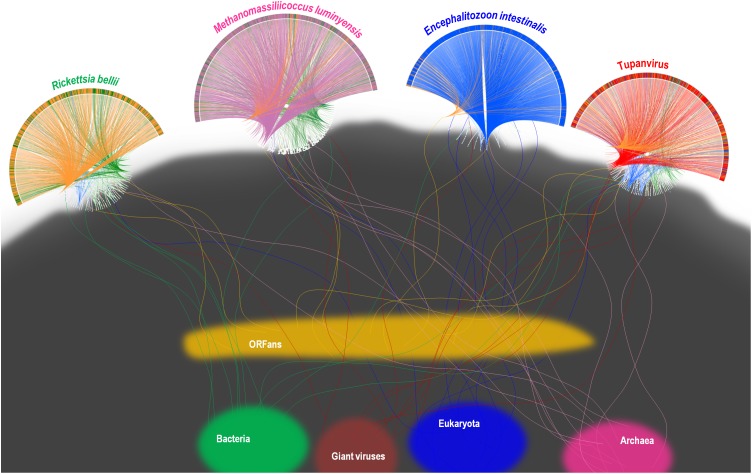
Representation as a rhizome of the genetic evolution for four current intracellular parasites of the four TRUCs of microbes with a comparable genome size, including *Rickettsia bellii* (a bacterium), *Methanomassiliicoccus luminyensis* (an archaeon), *Encephalitozoon intestinalis* (a microbial eukaryote), and Tupanvirus soda lake (a mimivirus). Rhizomes consist in a representation of genome evolution and mosaicism that takes into account that genes and intragenic sequences do not have the same evolutionary history, being proposed as better paradigm of genetic evolution than phylogenetic trees. The genomes of each of the four represented current microorganisms harbor mixtures of sequences of different origins. Sequences corresponding to current bacteria, Archaea, eukaryota, giant viruses, and to ORFans are colored in green, purple, blue, red, and orange, respectively. Rhizomes of the genomes of Tupanvirus and *Methanomassiliicoccus luminyensis* were adapted from same representations than representations from Levasseur et al. (2017) and Abrahao et al. (2018), respectively, licensed under CC BY 4.0 (https://creativecommons.org/licenses/by/4.0/) and CC-BY-NC (https://creativecommons.org/licenses/by-nc/4.0/), respectively (see legend to Figure 9).

A substantial genome mosaicism, consisting of genomes composed by genes with sequences suggesting different evolutionary origins and histories, was observed for representatives of the four TRUCs, including *R. bellii*, *M. luminyensis*, *E. intestinalis*, and Tupanvirus (Figure 9). This mosaicism was particularly predominant in the Tupanvirus genome as described previously (Abrahao et al., 2018), with 51, 11, 8, and 0.2% of its genes best matching with viruses, eukaryota, bacteria, and archaea, respectively, but it was a shared feature of the three non-eukaryotic microorganisms. This illustrates that a rhizome is the most appropriate representation of the evolutionary history at a genome scale, as individual genes can have distinct and distant origins (Raoult, 2010). Such representation notably takes into account introgressive descent as a result of lateral sequence transfers. Moreover, it appears that genes themselves may be subject to lateral sequence transfer rearrangements (through gene conversion), as shown here for the case of the methionyl-tRNA synthetase encoding gene of the four microorganisms (Figure 10). Indeed, 40 amino acid-long fragments of these genes alternately found as best hits, apart from relatives from the same family or genus, sequences from archaea, bacteria, eukaryota, or viruses. Such a gene sequence mosaicism was particularly broad for Tupanvirus and *M. luminyensis*. For the case of Tupanvirus soda lake, 15, 3, 2, and 1 methionyl-tRNA synthetase gene fragments found as best hits an eukaryote, a virus, a bacterium and an archaeon, respectively. This was also remarkably exemplified with the case of the glutaminyl-tRNA synthetase of Klosneuvirus, a mimivirus relative (Schulz et al., 2017). Indeed, fragments of this glutaminyl-tRNA synthetase gene showed a mixture of sequences from eukaryotes, bacteria and of unknown sources, or of sequences retrieved from metagenomes, in particular those of Antarctic dry valleys (Abrahao et al., 2018). These findings make the notion of gene lateral transfer obsolete, as sequences, rather than genes, are transferred (Merhej et al., 2011). Thus, the source of a gene may be better defined by a rhizome than by a tree, as previously proposed for organisms (Raoult, 2010) (Figure 11). Examples of chimeric genes have been previously described. Thus, ORF13 of the Sputnik virophage encodes a primase-helicase whose N-terminal region is of archaea-eukaryotic source and C-terminal portion was inferred to originate from giant viruses (La Scola et al., 2008). In the fern *Adiantum capillus-veneris*, a chimeric photoreceptor was identified that may have been critical in the divergence and rise of some fern species under low luminosity environments (Kawai et al., 2003). More broadly, it has been described that the creation of novel chimeric genes, referred as chimeric nuclear symbiogenetic genes (S-genes), occurred during eukaryogenesis through the fusion of bacterial and archaeal genes; this gave rise in early eukaryotes to novel chimeric proteins with central functions (Meheust et al., 2018). These data confirm and expand to genes the concept that no single tree can define the chimeric nature of genomes, as genes themselves are mosaics (Dagan and Martin, 2006; Merhej et al., 2011). As a consequence, trees made with homologous sequences make no sense if not all fragments of these sequences have a common source. Phylogeny reconstructions based on concatenated genes are still worse when the trees built based on the separate genes do not have the same topology, because they consist in mixing sequences from different, and eventually very distant, origins.

### Definition Criteria for Giant Viruses or Megavirales

As shown in Table 1, giant viruses exhibit unique phenotypic and genotypic features that differentiate them from ‘classical’ viruses, indicate their much greater complexity, and bring them close to small micro-organisms. These characteristics can be classified as follows: (i) Giant sizes of the virions and their genomes. (ii) Complexity, with presence in virions of dozens of proteins, and of messenger RNA. (iii) Presence of translation components unique among viruses; in this view, the recent characterization of klosneuviruses (Schulz et al., 2017) and tupanviruses (Abrahao et al., 2018) has led to a considerable expansion of the set of such translation components. Notably, the tupanvirus isolates encode for 67–70 tRNA, 20 aminoacyl tRNA-synthetases, and 11 translation factors. (iv) Presence of a specific mobilome in mimiviruses that includes virophages, transpovirons, introns, and endonucleases (Desnues et al., 2012), as well as MIMIVIRE, a defense system against virophages (Levasseur et al., 2016b; Dou et al., 2018). (v) Based on phylogenetic, phyletic, and protein fold superfamilies analyses, delineation of a fourth group of micro-organisms comprised by giant amoebal viruses alongside bacterial, archeal and eukaryotic microbes, and evidence of an archaic origin (Boyer et al., 2010; Sharma et al., 2014; Nasir and Caetano-Anollés, 2015). Moreover, the recent comparison of the genomes of a fossil and a modern pithovirus highlighted that giant viruses evolve with a mutation rate estimated to be lower than that of RNA viruses and comparable to those determined for bacteria and archaea, and by classical mechanisms of evolution, including through long-term fixation of genes that are acquired by horizontal gene transfer (Levasseur et al., 2016a).

Giant viruses of amoebae certainly exhibit several criteria that are hallmarks and definition criteria of viruses. These include the occurrence of an eclipse phase during their replicative cycle, an obligatory replication into host cells, and the presence of a capsid (Lwoff, 1957; La Scola et al., 2003; Raoult and Forterre, 2008). Nevertheless, regarding the capsid, pandoraviruses, pithoviruses, mollivirus, and cedratviruses have virions surrounded by a tegument-like structure and no known capsid morphology (Philippe et al., 2013; Yutin and Koonin, 2013; Legendre et al., 2014, 2015). Pandoraviruses do not have a recognizable capsid-encoding gene, pithoviruses have a barely identifiable capsid-encoding gene, while capsid proteins are detected in Mollivirus virions but they are not part of the virion structure. Other giant virions with an ovoid or spherical shape such as cedratviruses and Orpheovirus are also devoid of a morphology resembling those provided by known capsids. An atypical capsid structure was previously described for Megavirales representatives. Thus, most poxviruses have brick-shaped virions, the capsid precursors being assembled following icosahedral symmetry and the final shape being reached after proteolytical cleavages (Condit et al., 2006), and ascoviruses harbor allantoid capsids (Federici et al., 1990).

Moreover, although giant viruses of amoebae share phenotypic and genotypic features with cellular microorganisms, they were described to lack key cellular hallmarks. A first one consists in proteins involved in the production of energy. This might not be strictly true as tupanviruses harbor genes encoding a putative citrate synthase (Abrahao et al., 2018), and the genome of a distant mimivirus relative (Tetraselmis virus 1) that infects a green alga was shown to harbor key fermentation genes (a pyruvate formate-lyase and a pyruvate formate-lyase activating enzyme) that might ensure energy requirements (Schvarcz and Steward, 2018). A second one consists in ribosomal DNA and proteins, which are absent from giant viruses. Nevertheless, two distinct copies of an 18S rRNA intronic region were recently described in tupanviruses (Abrahao et al., 2018). These sequences were found to be highly expressed, and led to detect similar 18S rRNA intronic region in the majority of other mimivirus genomes. A third cellular hallmark that lacks in giant viruses of amoebae is binary fission as multiplication mechanism.

Conversely, it must be also considered that some bacteria display viral specific features and also lack hallmark features of cellular microorganisms. Numerous bacteria are indeed obligatory intracellular parasites. Moreover, some small cellular microorganisms such as *Carsonella ruddii* lack a comprehensive ATP generation machinery and, in addition, have a not comprehensive set of ribosomal proteins and aminoacyl-tRNA synthetases (Nakabachi et al., 2006; Tamames et al., 2007). Other cellular microorganisms, such as *Chlamydia* spp. (Abdelrahman et al., 2016; Bou Khalil et al., 2016), *Ehrlichia* spp. (Zhang et al., 2007), and *Babela* sp. (Pagnier et al., 2015) have no *bona fide* binary fission step during their multiplication. These data highlight that both classical viruses and cellular microorganisms can lack one or several pillar defining features. Finally, while a few viruses, including pandoraviruses, are devoid of capsid (Philippe et al., 2013; Koonin and Dolja, 2014), two classes of icosahedral compartments exist in bacteria and archaea that resemble to viral capsids: they include encapsulin nanocompartments structurally similar to and possibly derived from major capsid proteins of tailed bacterial and archaeal caudaviruses, and microcompartments present in bacteria (including cyanobacteria and many chemotropic bacteria) that encapsulate enzymes involved in metabolic pathways (Tanaka et al., 2008; Krupovic and Koonin, 2017).

## Conclusion And Perspectives

Viruses have long been considered as parasitic entities invisible by light microscopy and with a limited repertoire of genes (Raoult and Forterre, 2008). The fact that they are devoid of ribosomal genes has confined them outside of the “tree of life.” Giant viruses of amoebae have undermined this paradigm due to their characteristics that are, at the scale of classical viruses, outstanding (Raoult et al., 2007; Sharma et al., 2016). Phylogenies that were constructed here based on three ancient genes, including RNAP1/2 and DNA polymerase, delineate a fourth TRUC of microbes, as previously reported (Boyer et al., 2010; Sharma et al., 2014, 2015b). Hierarchical clustering performed using a set of informational COGs also shows a fourth independent branch alongside the three cellular branches. Because the tree of proteomes provides a more global and conserved phylogenomic view of protein domain composition in proteomes, their topologies can differ from single-gene based phylogenies that can independently indicate different evolutionary histories. However, here, the four branch topology was maintained in both sequence and structure based trees.

With the recent expansion of the proposed order Megavirales, the number of genes that are shared by these viruses and cellular organisms has shrunk, making it more difficult to build a fourth branch. Nevertheless, among the genes that still show a monophyly are polymerases, which were shown to be among the most ancient protein fold superfamilies (Nasir and Caetano-Anollés, 2015). The ancestrality of conserved genes such as the RNA polymerases, which are suspected to be more ancient than the ribosome (Nasir and Caetano-Anollés, 2015), highlights that evolution can be the result of structural constraints. This concept was described by Gould and Lewontin who used San Marco Cathedral’s spandrels to illustrate that adaptation through selection cannot comprehensively explain the evolution of genomes, and that biological constraints have to be considered (Gould and Lewontin, 1979). The structural, functional and evolutionary units of proteins are the structural domains, highly compact and recurrent segments of the molecules that often combine with others to perform major molecular and cellular tasks (Caetano-Anollés et al., 2009). Domains are evolutionarily highly conserved since they are defined by three-dimensional (3D) structural folds rather than amino acid sequences (Illergard et al., 2009). A rough estimate of evolutionary change suggests that a new fold structure takes millions of years to unfold, while a stable new sequence appears on Earth at least once every microsecond (Caetano-Anollés et al., 2009). In addition, hairpin-forming palindromes, which are possible primordial functional RNAs, are widely distributed among living entities, and they were found to be represented in giant viruses and virophages (Seligmann and Raoult, 2016). Short hairpin structures exist in the genomes of Mimivirus and the Sputnik virophage that may be involved in determining the polyadenylation site of transcripts (Byrne et al., 2009; Claverie and Abergel, 2009). While viral diversification appears fundamentally tailored by reductive evolution, the enrichment of viral genomes with primordial superfamilies of structural domains provides a strong support to the development of the viral proteome core prior to the inception of the ribosome but after the appearance of synthetase-like proteins capable of specific aminoacylation of tRNA molecules (Nasir and Caetano-Anollés, 2015). This could explain the existence of remnants of the translation machinery, the number of which has recently expanded considerably through the isolation of tupanviruses (Abrahao et al., 2018) and the assembly of klosneuvirus genomes (Schulz et al., 2017). As a matter of fact, it is unlikely that there has been a gradual and random acquisition of such large numbers of translation components in giant viruses, such as in mimiviruses, without using it. Hence, this translation machinery might have been acquired in a single step, or, alternatively, might have originated with giant viruses.

The classification of microbes, including the giant viruses, is more realistically based on their genomic content, which reflects their lifestyle, rather than on the phylogenies of supposedly representative genes, which may be confusing because of their mosaicism. This mosaicism results from sequence (and not gene) exchanges occurring during billion years of interactions between emerging lineages or organisms, and is particularly frequent between sympatric microorganisms (Moliner et al., 2010; Raoult and Boyer, 2010). Indeed, microorganisms that encounter and multiply or replicate in same biological niches are particularly prone to exchange nucleic acid sequences. This is well-suggested by the case of *Acanthamoeba* spp. that can be infected concomitantly by several amoeba-resistant microorganisms including intracellular bacteria and giant viruses with significantly larger repertoires than other related organisms (Moliner et al., 2010; Raoult and Boyer, 2010). Genes evolve by point mutations, but also by fusion, shuffling and fission of genetic fragments, which likely produce gene sequences that are mosaics (Long et al., 1999; Meheust et al., 2018; Pathmanathan et al., 2018). Such chimeric genes have been described in several studies (Ben et al., 2008; Merhej et al., 2011; Meheust et al., 2018), and we found here hints of such gene sequence mosaicism. In addition, many of the genes studied here encode for multi-domain proteins, which makes them mosaics of domains of different ages and histories. The phylogenomic tree reconstructed from domain structures that we describe here disentangles evolutionary histories because each domain becomes a separate phylogenetic character used to build the tree of proteomes. We note however that structural domains and their complex 3D topologies are also built from smaller module-like pieces of arrangements of helix, strand and turn segments (e.g., αα-hairpins, ββ-hairpins, βαβ-motifs) that act as evolutionary building blocks. Recent studies identified combinable (Goncearenco and Berezovsky, 2015) and no-combinable (Alva et al., 2015) ‘loop’ modules of these kinds. In fact, we recently studied the evolutionary combination of loops in domains by generating networks of loops and domains and by tracing their evolution along a timeline of billions of years (Aziz et al., 2016). We uncovered remarkable patterns such as the existence of two functional ‘waves’ of innovation associated with the ‘p-loop’ and ‘winged helix’ general domain structures, the preferential recruitment of ancient loops into new domain structures, and a pervasive network tendency toward hierarchical modularity. Given this difficult ‘mosaic’ problem that affects the sequences of genes and demands phylogenetic dissection, it is interesting to observe here that the tree of proteomes and the trees reconstructed from central genes provided a same overall phylogenetic insight of four TRUCs.

In summary, we highlight here the quantum leap that exists between classical and giant viruses. Our analyses confirm previous evidence of the existence of a fourth TRUC of life that includes viruses, and highlight its ancestrality and mosaicism. Results suggest that best representations for the evolution of giant viruses and cellular microorganisms are rhizomes, and, beyond, that mosaicism has to be considered at the genome (gene content) level but, more generally, at the gene and sequence level. Giant viruses may be represented as comprised by an evolutionary core inferred from highly conserved protein fold structures and gene sequences of very central and ancient proteins, surrounded by a larger and more dynamic gene complement characterized by genome and gene sequence mosaicisms. Such an abductive path as we use, which is based on phenotypic observations, is propitious to provide novel insight on microbial evolution. The “Fourth TRUC” club should, beyond any doubt, continue to expand in the near future, which may be boosted by using new amoebae as co-culture supports and by implementing high-throughput isolation strategies (Khalil et al., 2016). These giant viruses, as new biological entities, should continue to challenge previous paradigms, and a first step is to describe extensively these parasitic microbes without ribosomes.

## Author Contributions

DR, PC, PP, GC-A, BLS, and AL designed the experiments. PC, AL, GC-A, and DR wrote the manuscript. PC, AL, VS, AN, and GC-A performed the experiments. All authors analyzed the data and reviewed the manuscript.

## Conflict of Interest Statement

The authors declare that the research was conducted in the absence of any commercial or financial relationships that could be construed as a potential conflict of interest.

## Footnotes

1 ftp://ftp.ncbi.nih.gov/genomes/Viruses/

2 http://tree.bio.ed.ac.uk/software/figtree/

3 http://circos.ca/
