# Supplementary material for: Ancestrality and Mosaicism of Giant Viruses Supporting the Definition of the Fourth TRUC of Microbes
Source: Front Microbiol. 2018 Nov 27;9:2668. doi: 10.3389/fmicb.2018.02668 (PMC6277510; doi:10.3389/fmicb.2018.02668)
Supplement: Supplementary file 4 [file Data_Sheet_1.docx]

**SUPPLEMENTARY MATERIAL**

**Supplementary Table S1. List of the viruses that were used in the present analysis.**

**Supplementary Table S2. List and accession no. of gene sequences used in the present work.**

**Supplementary Table S3. List of whole coordinate information for building the PCoA plot of Figure 3.**
